# Global Prevalence of Chronic Kidney Disease – A Systematic Review and Meta-Analysis

**DOI:** 10.1371/journal.pone.0158765

**Published:** 2016-07-06

**Authors:** Nathan R. Hill, Samuel T. Fatoba, Jason L. Oke, Jennifer A. Hirst, Christopher A. O’Callaghan, Daniel S. Lasserson, F. D. Richard Hobbs

**Affiliations:** 1 Nuffield Department of Primary Care Health Sciences, University of Oxford, Oxford, United Kingdom; 2 Nuffield Department of Clinical Medicine, University of Oxford, Oxford, United Kingdom; Mario Negri Institute for Pharmacological Research and Azienda Ospedaliera Ospedali Riuniti di Bergamo, ITALY

## Abstract

Chronic kidney disease (CKD) is a global health burden with a high economic cost to health systems and is an independent risk factor for cardiovascular disease (CVD). All stages of CKD are associated with increased risks of cardiovascular morbidity, premature mortality, and/or decreased quality of life. CKD is usually asymptomatic until later stages and accurate prevalence data are lacking. Thus we sought to determine the prevalence of CKD globally, by stage, geographical location, gender and age. A systematic review and meta-analysis of observational studies estimating CKD prevalence in general populations was conducted through literature searches in 8 databases. We assessed pooled data using a random effects model. Of 5,842 potential articles, 100 studies of diverse quality were included, comprising 6,908,440 patients. Global mean(95%CI) CKD prevalence of 5 stages 13·4%(11·7–15·1%), and stages 3–5 was 10·6%(9·2–12·2%). Weighting by study quality did not affect prevalence estimates. CKD prevalence by stage was Stage-1 (eGFR>90+ACR>30): 3·5% (2·8–4·2%); Stage-2 (eGFR 60–89+ACR>30): 3·9% (2·7–5·3%); Stage-3 (eGFR 30–59): 7·6% (6·4–8·9%); Stage-4 = (eGFR 29–15): 0·4% (0·3–0·5%); and Stage-5 (eGFR<15): 0·1% (0·1–0·1%). CKD has a high global prevalence with a consistent estimated global CKD prevalence of between 11 to 13% with the majority stage 3. Future research should evaluate intervention strategies deliverable at scale to delay the progression of CKD and improve CVD outcomes.

## Introduction

Chronic kidney disease (CKD) is associated with age-related renal function decline accelerated in hypertension, diabetes, obesity and primary renal disorders. [1] Cardiovascular disease (CVD) is the primary cause of morbidity and mortality where CKD is regarded as an accelerator of CVD risk and an independent risk factor for CVD events. [2] There is a graded inverse relationship between CVD risk and glomerular filtration rate (GFR) that is independent of age, sex and other risk factors. [3–6] Decreased renal function is a predictor of hospitalisation [1, 2], cognitive dysfunction [7] and poor quality of life. [8, 9] The healthcare burden is highest in early stages due to increased prevalence, affecting around 35% of those over 70 years. [10]

CKD is defined by indicators of kidney damage—imaging or proteinuria (commonly using albumin to creatinine ratio, ACR)—and decreased renal function (below thresholds of GFR estimated from serum creatinine concentration). [11, 12] Current recommendations by Kidney Outcomes Quality Initiative (KDOQI) and National Institute for Health Excellence (NICE) [11, 12] are to use serum creatinine concentration to estimate GFR (eGFR) and transform it using the Chronic Kidney Disease Epidemiology Collaboration (CKD-EPI) equation. [13] CKD-EPI replaces the Modification of Diet in Renal Disease (MDRD) equation [14] as a more accurate predictor of clinical risk [15] and both these equations correct for selected non-renal influences (age, race, gender).

CKD can be classified into five stages using KDOQI [11] guidelines using thresholds of eGFR within the CKD range and/or evidence of structural renal changes e.g. proteinuria. NICE have suggested that stage 3 be subdivided into 3a and 3b reflecting increasing CVD risk. [12] The largest stage of CKD, with over 90% of cases, has been estimated from a UK retrospective lab audit study to be CKD stage 3 with 84% stage 3a (GFR of 45 to 59 ml/min/1·73m^2^) and 16% stage 3b GFR of 30 to 44 ml/min/1·73m^2^. [16]

Changes over time in CKD prevalence are contentious. Data from the American National Health and Nutrition Examination Survey demonstrate that in the period 1999 to 2004 the prevalence of CKD stages 1 to 4 increased significantly when compared to the survey period 1988 to 1994 (13·1 versus 10·0%). [4, 17, 18] While this high (and rising ^4^,) prevalence is in part due to the ageing population, it is also associated with increases in hypertension and diabetes mellitus[1]. However, conversely a UK manuscript published in 2014 examined nationally representative cross-sectional studies within the UK and found that the prevalence estimates reported declined over time. [19]

CKD is recognised as having changed from a subspecialty issue to a global health concern. [20] The authors, therefore, sought to determine the global prevalence of CKD according to KDOQI criteria in published observational studies in the adult general population, by a systematic review and meta-analysis.

## Materials and Methods

### Search strategy and selection criteria

The protocol has been published (PROSPERO: CRD42014009184) and conducted in accordance with the Meta-analysis Of Observational Studies in Epidemiology guidelines [21]. Search strategy was discussed with a librarian for optimum inclusion sensitivity. An early consensus panel on the search results expanded the criteria to include additional general populations not identified originally (e.g. laboratory based large population studies). The librarian performed iterative searches using the following repositories for published observational studies: Medline/PubMed, Embase, CINAHL, the Cochrane Register for Controlled Trials (CENTRAL), LILACS, SciELO, clinicaltrials.gov, WHO ICTRP. They used the Cochrane Collaboration’s Highly Sensitive Search Strategy to optimize results. [22] The search strategy for clinicaltrials.gov was Condition = (“kidney disease” OR “kidney failure” OR “kidney insufficiency” OR “kidney function” OR “kidney dysfunction” OR “renal disease” OR “renal failure” OR “renal insufficiency” OR “renal function” OR “renal dysfunction”) AND Outcome = prevalence. The reference lists of other systematic reviews on prevalence of CKD were searched for potentially relevant articles. All databases were searched from inception to the 1st September 2014.

### Study selection and data extraction

Original peer-reviewed publications were selected by two authors (NH, SF) if they included: a >500 people, conducted from year 2000+, used MDRD/CKD-EPI formula, reported CKD prevalence using KDOQI criteria and were in the general population (even if limited—e.g. aged >65). Studies were excluded if they had no criteria for diagnosis of CKD, did not include prevalence, were in a specialist restricted population (e.g. acute hospital patient cohort, nursing home), were an audit of existing results already included or if there was a more recent updated study. Translations were sought for non-English articles.

Data extraction was with standardised forms by two independent reviewers (NH, SF) disagreement was resolved by adjudicator (DL). Data included quality assessment, prevalence of CKD, method used to calculate eGFR, study setting: year, country, the population, gender split, age, and so on. Authors of relevant articles were contacted to provide additional information whenever necessary and references of selected articles were hand searched for additional articles. The KDOQI definition of CKD stages was used [11] and the method, calibration and traceability of the creatinine assessment extracted.

### Statistical analysis and quality assessment

CKD prevalence was defined by the studies as being calculated for Stages 1 to 5 (eGFR & ACR) or Stages 3 to 5 (eGFR alone). 95% confidence intervals (95%CI) were calculated for each prevalence value. Meta-analyses were performed in Stata version 14. A procedure for pooling proportions in the meta-analysis of multiple studies study was used and the results displayed in a forest plot. The 95%CI’s are based on score(Wilson) procedures [23]. Heterogeneity was quantified using the I-squared measure, The I^2^ heterogeneity was categorised as follows: <25% low, 25 to 50% moderate and >50% high [24]. A Freeman-Tukey Double Arcsine Transformation [25] was used to stabilise the variance prior to calculation of the pooled estimates. Random effects models were selected for the meta-analyses with the assumption that CKD prevalence by country would be variable.

Subgroup analysis was undertaken by country, geographic region, age and gender. Geographic regions were defined based on the geographic proximity of the country the studies occurred in and the possible similarity in the ethnicity of the populations. Meta-regression was weighted by number of subjects unless otherwise specified [24]. Random effects meta-regressions using aggregate level data for CKD prevalence, study year, participant characteristics and co-morbidities were performed.

Methodological quality was assessed by one reviewer (NH) defined as adherence to STROBE (Strengthening the Reporting of Observational Studies in Epidemiology Statement) recommendations. [26] The STROBE 22-point checklist was used to score each manuscript, items that had subdivision recommendations scored a point for each. Serum creatinine reporting quality was assessed by two reviews (NH & SF)—traceability of assay, number of measurements per patient, assay method used, and calibration of assay. A combined quality score was generated from methodological quality- as measured by STROBE adherence- and serum creatinine reporting quality. The weighting was arbitrarily chosen to be two-thirds STROBE adherence and one-third creatinine reporting. To assess bias, quality was used to weight CKD prevalence values in a meta-analysis.

Sensitivity analyses were undertaken to investigate the individual study influence and of limited populations (high altitude, single site of recruitment in rural area, single site of recruitment in urban area, laboratory audit, age by decile, or age restricted), studies that used age adjusted prevalence and using only high quality studies—quality score threshold of 56% (mean quality). Further sensitivity analyses were undertaken using studies that examined IDMS traceable creatinine only, studies that used double measuring of creatinine, studies that achieved two or more of the serum creatinine reporting quality items, and studies that used different eGFR equations (CKD-EPI or MDRD).

## Results

**Fig 1:**
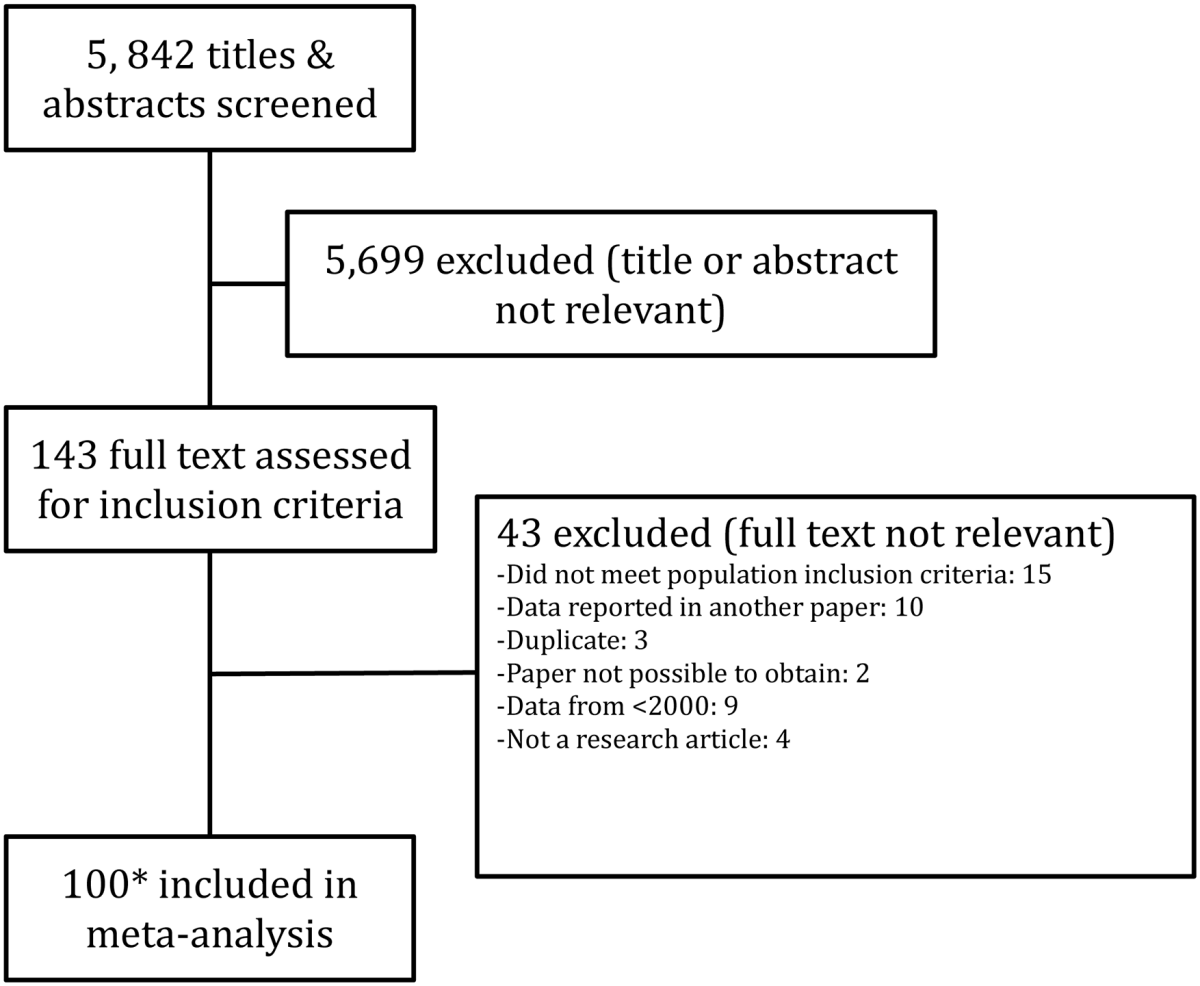
Systematic review flow diagram of manuscripts screened, excluded and included in meta-analysis. *112 Populations from 100 manuscripts as some manuscripts reported on more than one population or split their populations prior to analysis.

The search yielded 5,842 articles after duplicates had been removed and 143 articles were assessed relevant for the review by title and abstract. Forty-three were excluded on full manuscript assessment. A detailed review and data extraction was conducted on 100 manuscripts (covering 112 populations), Fig 1. No additional studies were identified by examining reference lists. All studies that were included were published after the introduction of the KDOQI 2002 CKD definition guidelines [11].

China had the highest number of population samples with seventeen. [27–43] Numbers of participants ranged from 778 in a USA cohort [44] to 1,120,295 in a USA laboratory audit [2]. The S1 Appendix Study Table details the relevant details of all studies and populations.

### Prevalence

**Fig 2:**
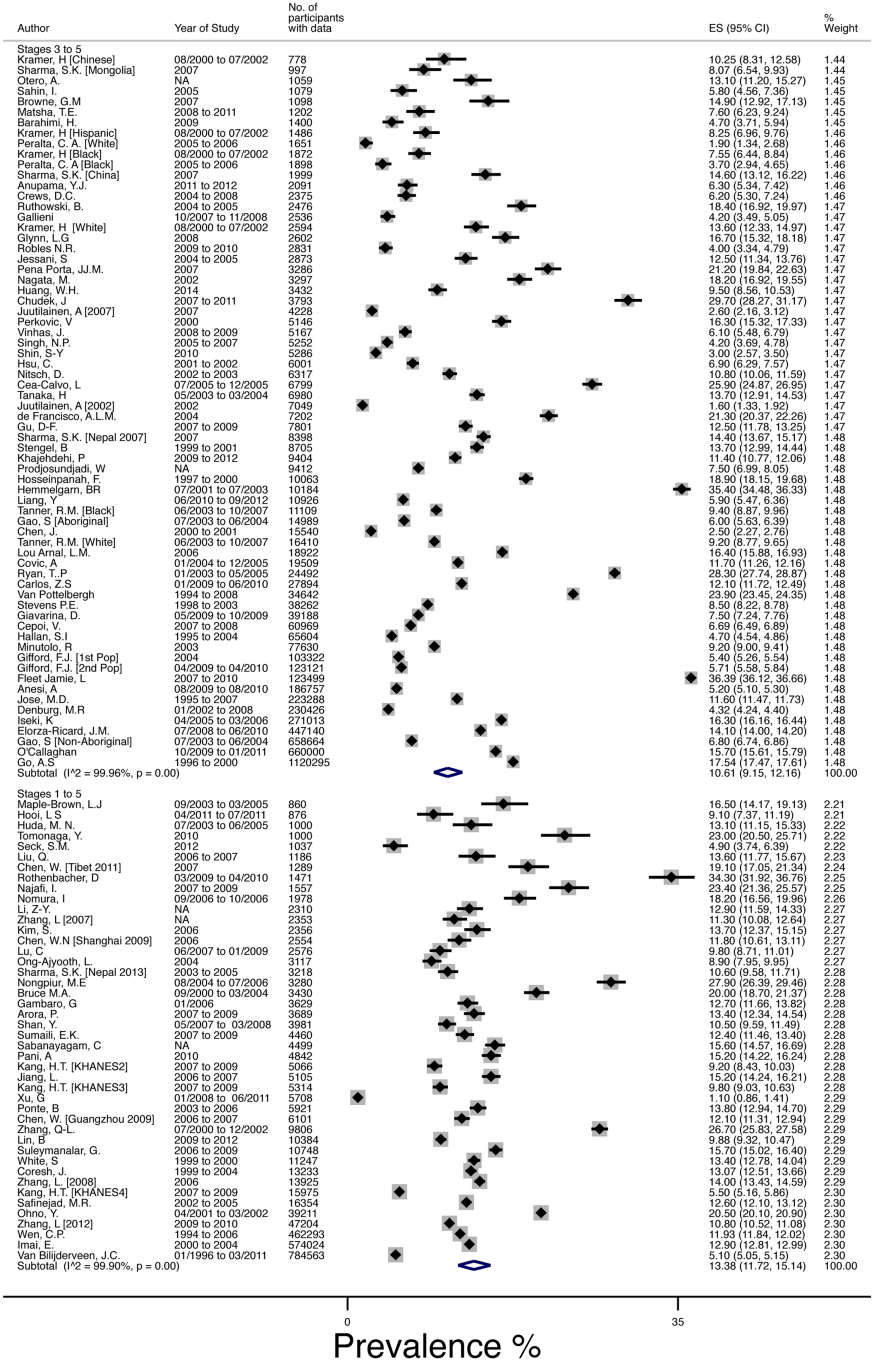
Meta Analysis of CKD prevalence using random effects model, weighted by standard error of the mean estimates. Studies are ordered by number of participants and split by whether the report 3 stages of CKD (“Three”) or five stages of CKD (“Five”).

The mean(95%CI) global prevalence of CKD was 13·4%(11·7–15·1%), I^2^ = 99.9%, for the forty-four populations that measured prevalence by all 5 stages (1 to 5) [4, 28, 29, 32, 33, 35–38, 40–43, 45–73], and 10·6%(9·2–12·2%),I^2^ = 100%, in the sixty-eight populations [2, 10, 27, 30, 31, 34, 39, 44, 74–123] measuring Stages 3 to 5, Fig 2.

CKD prevalence breakdown was provided in seventy-four populations. [2, 4, 10, 27–29, 32, 35, 37–43, 46, 47, 50–56, 60, 63, 65–71, 73–84, 86–91, 98–102, 106–111, 113, 116, 118, 121, 122] The 1 to 5 stages mean CKD prevalence was higher (13·4% vs. 11·0%). The breakdown by stage using all available data was Stage-1 (eGFR>90+ACR>30): 3·5%(2·8–4·2%); Stage-2 (eGFR 60–89+ACR>30): 3·9%(2·7–5·3%); Stage-3 (eGFR 30–59): 7·6%(6·4–8·9%); Stage-4 = (eGFR 29–15): 0·4%(0·3–0·5%); and Stage-5 (eGFR<15): 0·1%(0·1–0·1%). Separate reporting of Stage 3a/3b was not possible due to lack of reporting. Sensitivity analyses determined that no individual study or group of studies (limited populations—i.e. laboratory audits, age restricted, single site recruitment—, age adjusted prevalence, etc.) were suspected of excess influence on the prevalence estimates. Further, there was no difference between studies that reported using the higher quality IDMS traceable assay and those that did not.

#### Effect of Age, Hypertension, BMI, Obesity, Diabetes, Smoking

Univariate meta-regressions of CKD prevalence and covariates were undertaken. Mean population age, given in 94 of 112 populations, was significantly associated (β = 0·4%, p<0·001, R^2^ = 25·5), as was prevalence of diabetes (n = 82, β = 0·16%, p = 0·006, R^2^ = 8·0), prevalence of hypertension (n = 75, β = 0·15%, p = 0·002, R^2^ = 11·4) but not average BMI or prevalence of obesity. Smoking (n = 60) was negatively associated with CKD prevalence (an increase of smoking status was associated with a decreased prevalence of CKD (β = -0·14 p = 0·07, R^2^ = 4·2).

**Fig 3:**
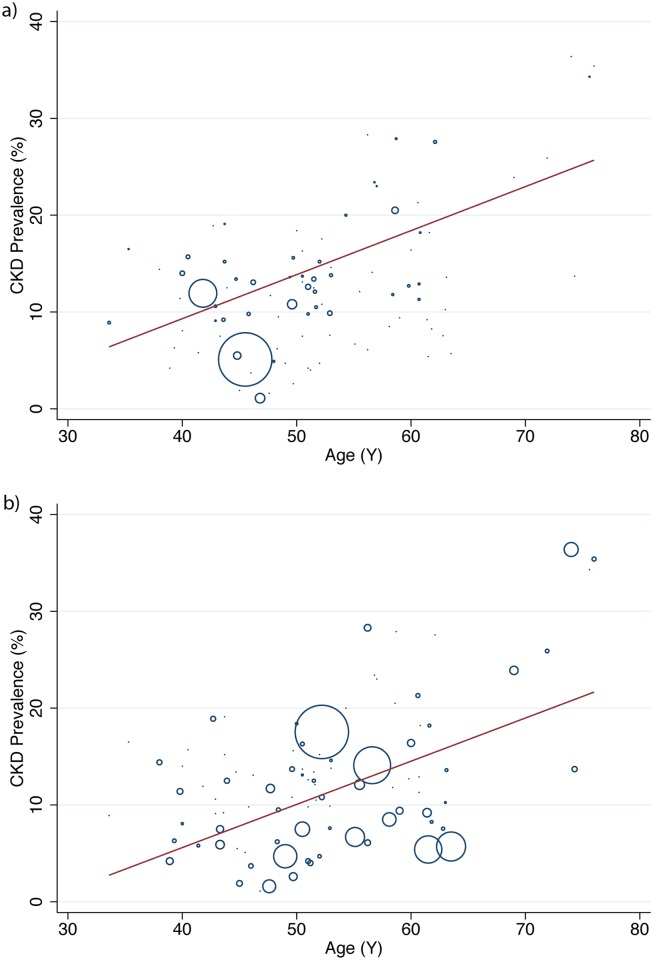
Meta Regression of CKD Prevalence and mean sample population age (a) Studies reporting stages 1 to 5 (b) Studies reporting stages 3 to 5. Each circle represents a study prevalence estimate with the size denoting the precision of the estimate.

Prevalence of CKD increased with age, Fig 3. To determine an estimated prevalence for each age the sample population was divided by mean age into deciles. Studies measuring 5 stages of CKD mean(95%CI) were—30s: 13·7%(10·8, 16·6%), 40s: 12·0%(9·9, 14·1%), 50s: 16·0%(13·5, 18·4%), 60s: 27·6%(26·7, 28·5%), 70s: 34·3%(31·9, 36·7%). Studies measuring stages 3 to 5–30s: 8·9%(4·7, 13·1%), 40s: 8·7%(6·9, 10·5%), 50s: 12·2%(9·8, 14·5%), 60s: 11·3%(8·1, 14·5%), 70s: 27·9%(16·40, 39·3%).

There were no significant differences in prevalence between groups of studies that adjusted for age compared to those that did not. Further, a sensitivity test found that older age restricted populations did not significantly change the estimated pooled prevalence for CKD, Stages 3 to 5 mean (95%CI) 10·2%(8·4–12·0%) vs. 10·6%(9·2–12·2%) and stages 1 to 5 mean (95%CI) 11·5%(9·3–13·9%) vs. 11·4%(9·4–13·1%). A sensitivity analysis examining glomerular filtration estimating equation was planned but only 12 studies used the CKD-EPI equation making the analysis unfeasible.

### Geography

CKD prevalence by geographical grouping was examined, Table 1. Geographical areas with more than one study were pooled using random effects models.

**Table 1:** Mean prevalence of CKD split by geographical region with 95% Confidence Intervals.

	Stage 1 to 5		Stages 3 to 5	
	N*	Prevalence (%)	N*	Prevalence (%)
**S Africa, Senegal, Congo**	5,497	8.66 (1.31, 16.01)	1,202	7.60 (6.10, 9.10)
**India, Bangladesh**	1,000	13.10 (11.01, 15.19)	12,752	6.76 (3.68, 9.85)
**Iran**	17,911	17.95 (7.37, 28.53)	20,867	11.68 (4.51, 18.84)
**Chile**	0	NONE	27,894	12.10 (11.72, 12.48)
**China, Taiwan, Mongolia**	570,187	13.18 (12.07, 14.30)	62,062	10.06 (6.63, 13.49)
**Japan, S Korea, Oceania**	654,832	13.74 (10.75, 16.72)	298,000	11.73 (5.36, 18.10)
**Australia**	12,107	14.71 (11.71, 17.71)	896,941	8.14 (4.48, 11.79)
**USA, Canada**	20,352	15.45 (11.71, 19.20)	1,319,003	14.44 (8.52, 20.36)
**Europe**	821,902	18.38 (11.57, 25.20)	2,169,183	11.86 (9.93, 13.79)

*N is number of participants in the sample estimate.

### Gender

Fifty-one studies reported sex-specific prevalence of CKD. [27–29, 32, 37–39, 44, 46, 48, 50, 52–55, 57, 58, 61, 64, 68–71, 78, 79, 82, 83, 85, 87, 92–94, 98, 100–103, 105, 106, 108, 110, 115, 119] Male mean (95%CI) CKD prevalence, for studies that defined 5 stages of CKD, was 12·8%(10·8–11·9%) and for studies that used stages 3 to 5 it was 8·1%(6·3–10·2%). Female CKD prevalence for studies that defined CKD by stages 1 to 5 was 14·6%(12·7–16·7%) and for studies that used stages 3 to 5 it was 12·1%(10·6–13·8%). Thirty-eight studies [27–29, 32, 37–39, 44, 46, 50, 55, 64, 69–71, 78, 79, 83, 85, 87, 92, 98, 100–103, 105, 106, 108, 110, 115, 119] reported that CKD was more prevalent in women than in men with the pattern reversed in thirteen studies. [39, 44, 48, 52–54, 57, 58, 61, 68, 82, 93, 94]

### Quality

The methodological quality of studies ranged from 32·1% [31] to 92·9%. [4, 68] No study complied completely with the STROBE guidelines and the mean(SD) quality was 69·6(12·5)%.

Quality of serum creatinine measurement was assessed. Two studies scored 100%- four methods. [115, 122] Thirty-six studies scored 0% [30–32, 34, 39, 40, 48, 49, 52, 57–60, 62, 64, 71, 72, 74, 76, 81, 82, 85, 87, 89, 92, 95, 102, 103, 105, 106, 109, 113, 116, 117], thirty-five studies scored 25%-one method-[2, 28, 29, 33, 35–38, 41, 43, 47, 51, 53, 61, 65, 67–70, 75, 77–79, 83, 84, 97–100, 108, 110, 114, 121], twenty-seven scored 50%-two methods-[4, 27, 42, 44, 46, 50, 54–56, 66, 74, 80, 88, 90, 101, 104, 107, 111, 112, 118, 119, 123] and ten scored 75%-three methods. [10, 45, 63, 73, 86, 91, 93, 94, 96, 120]

Sensitivity analyses determined no difference in the prevalence estimate of CKD when using only high quality studies, studies that used double measures of creatinine only or studies that had two or more factors for the measurement of creatinine.

## Discussion

CKD prevalence Stages 1 to 5 was 13·4% and 10·6% in stages 3 to 5. This systematic review is the first meta-analysis of CKD prevalence globally and provides a comprehensive overview of the current literature. These estimates indicate that CKD may be more common than diabetes, which has an estimated prevalence of 8·2%. [124] However, the reported prevalence of CKD varied widely amongst the studies and had high heterogeneity.

CKD was more prevalent in women than in men. Two-thirds of studies -that reported gender-specific CKD prevalence- determined higher prevalence in women. Women, in general, have less muscle mass than men and muscle mass is a major determinant of serum creatinine concentration. However, the GFR estimation equations adjust for gender differences, using a correction factor for women. These findings add to the existing literature that recognise a gender-specific difference between CKD prevalence. [125–127]However, these data cannot answer why this may occur. We can speculate that this finding may be partially explained by selection bias inherent within the studies due to a different age demographic for the two sexes. Alternatively it may be due to complex factors in the disease pathology that are not captured within the studies. Or that there is in fact more renal disease in men but the eGFR equations preferentially identify renal disease in women in the stage 3 zones.

Studies that were outliers in terms of reported results were of interest. Smoking was found to be negatively associated with CKD prevalence but this finding was negated when a single outlier was removed. The outlier [120] was a study in which smoking was defined as >100 cigarettes ever and thus 69·1% were smokers. A Spanish study [106] (n: 7202, Quality: 52%, CKD: 21·3%) reported 66.7% hypertension prevalence within the population compared with a global mean (from all other studies) of 31·1%. Hypertension was not defined any differently. Further, 31.5% of their sample population had diabetes and 31.1% were obese. The population was reported as unrestricted older population but although it was older than other studies (mean age 60·6yrs) these rates of co-morbidity are unexpected and were not explained. A number of studies had very high prevalence of CKD (>30%) the highest of these was a Canadian study (n: 123,499, Quality: 52%, CKD: 36·4%), a laboratory audit of patients over 65 years. The prevalence observed may be due to selection bias as the mean age of this cohort was 74 years, with 23% diabetes in the sample population, two factors associated with renal decline.

The geographical stratification of results revealed that developed areas such as Europe, USA, Canada and Australia had higher rates of CKD prevalence in comparison to areas where economies are growing such as sub Saharan Africa, India etc. With the exception of Iran that had similar high level of CKD prevalence possibly due dietary risks, high BMI, high systolic BP and co-morbid conditions within the country [128]. Although percentage prevalence was higher in more developed areas projected worldwide population changes will increase the absolute numbers of people in developing countries where the populations of elderly are increasing. This increase will exacerbate the double burden of dealing with communicable and non communicable disease in a developing economy[129].

Serum creatinine measurement bias was inherent in the majority of the studies. Serum creatinine concentrations are highly variable within individuals, up to 21% within a 2-week period. [130] NICE guidelines advise two measures of eGFR 3-months apart and within the last 12-months to minimise intra-individual variation. Not all countries have such guidelines only 5 manuscripts reported this in study design. Jaffe creatinine assay was the main method used but it is known to systematically overestimate serum creatinine to varying degrees. [131] Thirty-seven of the studied populations reported that they calibrated directly to the laboratory to minimize assay bias effect and twenty-seven studies used a minimally biased traceably assay (IDMS). A comparison of these studies to the remainder found no significant difference in prevalence estimates. A third of the studies (n = 36) made no mention of measures, traceability, or calibrations. It is further known that the MDRD equation systematically overestimates CKD in the general population [13] and the prevalence rates calculated may be lower. Estimated GFR is accepted as the most useful index of kidney function in health and disease, but an uncorrected, untraceable single measure inherently introduces noise and outliers into the dataset. This latter point has been very recently clarified as an epidemiological study in Morocco found that up to 30% of patients initially classified as CKD 3a using the MDRD formula had improved renal function over 12 months and therefore would not have a CKD diagnosis[132].

Estimation of GFR from serum creatinine is the clinical standard worldwide and the CKD the KDOQI diagnostic criteria[11] guidelines emphasise the importance of estimation of GFR rather than use of serum creatinine concentration. However, the 2002 KDOQI guidelines that the included studies reference have stimulated controversies and questions. In particular, there have been concerns that use of its definition of CKD has caused excessive false identification of CKD and that its staging system was not sufficiently informative about prognosis. A new KDIGO guideline was published in 2013 [133] that sought to address this with the splitting of the stage 3 category to emphasise the risks of mortality and other outcomes vary greatly between these groups and have further and further sub-stratified by the inclusion of urinary albuminuria. There is a limitation in our study in that unfortunately the analysis of stages 3a and 3b was not possible due to lack of reporting and studies using the previous KDOQI guidelines so no conclusions about whether the patients really have ‘disease’ rather than normal variation due to aging could be drawn.

Observational studies are individually subject to bias and residual confounding from unspecified sources but it is difficult to quantify how much bias and/or confounding. One study may report an effect size adjusted for several possible confounders; others may report the crude prevalence. The authors have sought to address this limitation by using STROBE quality weighting and creatinine quality factors and participant per study-weighted rates. Ideally future research should report the crude and adjusted rates based on multiple measures over time.

This systematic review and meta-analysis significantly extends existing systematic reviews in a number of ways. The search strategy allowed the detection of a large number of additional studies that had not been considered in previous systematic reviews. It increased the number of reference databases searched. The reviewers undertook to screen non-English publications through the use of translations. The studies included used the same definitions of CKD and used broadly comparable definitions for severity markers or related conditions (albuminuria, hypertension, diabetes and obesity). However, there are limitations due to the heterogeneity that arises from differences in age and sex distributions, use of creatinine assays, different sampling frames, inclusion criteria of general population based studies, and time period of the study. A proportion of the variation across studies may not be due to real differences in CKD prevalence. However, the authors did seek to provide a robust assessment of the quality and use this to determine a weighted global prevalence of CKD in the meta-analysis. The prevalence rates calculated highlight the likely numbers of people with CKD that may be of relevance to health care providers and national health programs with finite resources with which to address this epidemic.

CKD constitutes a major cost burden to healthcare systems worldwide. The high prevalence and the extensive existing evidence that intervention is effective in reducing CVD events demonstrates a need for national initiatives that will slow the progression to end stage renal disease and reduce CVD-related events in CKD patients.

This comprehensive meta-analysis of observational studies confirms that CKD has a high prevalence. Using CKD prevalence weighted by quality, using ‘High’ quality studies only and using studies weighted by number of participants consistently estimated a global CKD prevalence of between 11 to 13%. Future research should evaluate intervention strategies deliverable at scale to delay the progression of CKD and improve CVD outcomes. Evaluation of the roles of these interventions and the associated costs needs to be undertaken. CKD prevalence studies should report more detail on disease definitions and population demographics and state unadjusted as well as adjusted findings.

## Supporting Information

S1 Appendix Study Table—Summary descriptions of included studies (n = 100) and the populations (n = 112) within those studies. (DOCX)

S2 Appendix PRISMA Checklist—Preferred Reporting Items for Systematic Reviews and Meta-Analyses checklist. (DOC)
