# Supplementary material for: Global Prevalence of Chronic Kidney Disease – A Systematic Review and Meta-Analysis
Source: PLoS One. 2016 Jul 6;11(7):e0158765. doi: 10.1371/journal.pone.0158765 (PMC4934905; doi:10.1371/journal.pone.0158765)
Supplement: S1 Appendix — (DOCX) [file pone.0158765.s001.docx]

| **Author (ref) year of study** | **Country** | **Number of participants, setting (Urban, Rural, Mixed), age, BMI, gender** | **Co-morbidity (%): Diabetes, hypertension, proteinuria, obesity, smoking** | **Creatinine: Equation,**  **Double measure, method described?, calibrated?, traceable?** | **Quality** | **CKD Prevalence** | **CKD Stages (%)** | | | | |
| --- | --- | --- | --- | --- | --- | --- | --- | --- | --- | --- | --- |
|  |  |  |  |  |  |  | **1** | **2** | **3** | **4** | **5** |
| Anesi, A ([90](#_ENREF_90)) 08/2009 to 08/2010 | Italy | n=186757, setting:MIXED, mean age:NR, mean BMI:NR, 52% females. | DM:NR, HTN:NR, PU:NR, OB:NR, SMK:NR | Equation: MDRD Double: FALSE Method: TRUE Calibrated: FALSE Traceable: TRUE | 52% | Overall: 5.2% Male: NR Female: NR | NR | NR | 4.1 | 0.6 | 0.5 |
| Anupama, Y.J. ([81](#_ENREF_81)) 2011 to 2012 | India | n=2091, setting:RURAL, mean age:39.3, mean BMI:NR, 54.4% females. | DM:3.8, HTN:33.6, PU:2.9, OB:13.3, SMK:NR | Equation: MDRD Double: FALSE Method: FALSE Calibrated: FALSE Traceable: FALSE | 28% | Overall: 6.3% Male: NR Female: NR | NR | NR | 4.3 | 0.1 | 0.0 |
| Arora, P. ([48](#_ENREF_48)) 2007 to 2009 | Canada | n=3689, setting:MIXED, mean age:44.7, mean BMI:27.1, 50% females. | DM:6.3, HTN:16.3, PU:NR, OB:23, SMK:20.7 | Equation: MDRD Double: FALSE Method: FALSE Calibrated: FALSE Traceable: FALSE | 45% | Overall: 13.4% Male: 15.4% Female: 13.4% | NR | NR | NR | NR | NR |
| Barahimi, H. ([85](#_ENREF_85)) 2009 | Iran | n=1400, setting:RURAL, mean age:52, mean BMI:26, 68% females. | DM:3.4, HTN:13.7, PU:16.2, OB:NR, SMK:NR | Equation: MDRD Double: FALSE Method: FALSE Calibrated: FALSE Traceable: FALSE | 33% | Overall: 4.7% Male: 1.8% Female: 6.1% | NR | NR | NR | NR | NR |
| Browne, G.M ([88](#_ENREF_88)) 2007 | Ireland | n=1098, setting:MIXED, mean age:NR, mean BMI:NR, 55.1% females. | DM:7.8, HTN:NR, PU:13.2, OB:NR, SMK:47.4 | Equation: MDRD Double: FALSE Method: TRUE Calibrated: FALSE Traceable: TRUE | 76% | Overall: 14.9% Male: NR Female: NR | NR | NR | 14.5 | 0.4 | 0.0 |
| Bruc,e M.A. ([72](#_ENREF_72)) 09/2000 to 03/2004 | USA | n=3430, setting:MIXED, mean age:54.3, mean BMI:NR, 63.7% females. | DM:17.9, HTN:62.6, PU:12.5, OB:85, SMK:NR | Equation: MDRD Double: FALSE Method: FALSE Calibrated: FALSE Traceable: FALSE | 50% | Overall: 20% Male: NR Female: NR | NR | NR | NR | NR | NR |
| Carlos, Z.S ([78](#_ENREF_78)) 01/2009 to 06/2010 | Chile | n=27894, setting:MIXED, mean age:55.5, mean BMI:NR, 66% females. | DM:NR, HTN:NR, PU:NR, OB:NR, SMK:NR | Equation: MDRD Double: FALSE Method: TRUE Calibrated: FALSE Traceable: FALSE | 56% | Overall: 12.1% Male: 7.4% Female: 14.5% | NR | NR | 11.6 | 0.3 | 0.2 |
| Cea-Calvo, L ([105](#_ENREF_105)) 07/2005 to 12/2005 | Spain | n=6799, setting:MIXED, mean age:71.9, mean BMI:28.5, 53.6% females. | DM:27, HTN:72.9, PU:NR, OB:34.4, SMK:10.6 | Equation: MDRD Double: FALSE Method: FALSE Calibrated: FALSE Traceable: FALSE | 45% | Overall: 25.9% Male: 14.1% Female: 36.1% | NR | NR | NR | NR | NR |
| Cepoi, V. ([102](#_ENREF_102)) 2007 to 2008 | Romania | n=60969, setting:MIXED, mean age:55.1, mean BMI:NR, 55% females. | DM:NR, HTN:NR, PU:NR, OB:NR, SMK:NR | Equation: MDRD Double: FALSE Method: FALSE Calibrated: FALSE Traceable: FALSE | 24% | Overall: 6.69% Male: 3.7% Female: 9.09% | NR | NR | 6.2 | 0.1 | 0.1 |
| Chen, J. ([27](#_ENREF_27)) 2000 to 2001 | China | n=15540, setting:MIXED, mean age:NR, mean BMI:NR, NR females. | DM:NR, HTN:NR, PU:NR, OB:NR, SMK:NR | Equation: MDRD Double: FALSE Method: TRUE Calibrated: TRUE Traceable: FALSE | 55% | Overall: 2.5% Male: 1.4% Female: 3.8% | NR | NR | 2.4 | 0.1 | 0.0 |
| Chen, W. [Guangzhou 2009] ([28](#_ENREF_28)) 2006 to 2007 | China | n=6101, setting:URBAN, mean age:51.6, mean BMI:23.6, 67% females. | DM:5.5, HTN:19.2, PU:6.6, OB:30, SMK:18.8 | Equation: MDRD Double: FALSE Method: FALSE Calibrated: TRUE Traceable: FALSE | 58% | Overall: 12.1% Male: 10% Female: 13.9% | 4.1 | 4.8 | 2.8 | 0.3 | 0.1 |
| Chen, W. [Tibet 2011] ([70](#_ENREF_70)) 2007 | Tibet | n=1289, setting:MIXED, mean age:43.7, mean BMI:24.3, 72% females. | DM:2.9, HTN:38.8, PU:16.2, OB:16.7, SMK:12.4 | Equation: MDRD Double: FALSE Method: TRUE Calibrated: FALSE Traceable: FALSE | 58% | Overall: 19.1% Male: 18.5% Female: 22.7% | 12.8 | 4.6 | 1.1 | 0.3 | 0.3 |
| Chen, W.N [Shanghai 2009] ([29](#_ENREF_29)) 2006 | China | n=2554, setting:URBAN, mean age:58.4, mean BMI:NR, 64% females. | DM:12, HTN:53.1, PU:6.3, OB:4.6, SMK:NR | Equation: MDRD Double: TRUE Method: FALSE Calibrated: FALSE Traceable: FALSE | 49% | Overall: 11.8% Male: 10.8% Female: 12.4% | 2.4 | 3.6 | 5.5 | 0.3 | 0.0 |
| Chudek, J ([98](#_ENREF_98)) 2007 to 2011 | Poland | n=3793, setting:MIXED, mean age:NR, mean BMI:NR, 49.1% females. | DM:22.7, HTN:, PU:NR, OB:33, SMK:43.7 | Equation: CKD-EPI Double: FALSE Method: TRUE Calibrated: FALSE Traceable: FALSE | 60% | Overall: 29.7% Male: 27.5% Female: 32.1% | NR | NR | 19.8 | 1.1 | 0.2 |
| Coresh, J. ([4](#_ENREF_4)) 1999 to 2004 | USA | n=13233, setting:MIXED, mean age:46.2, mean BMI:28.1, 51.8% females. | DM:6.8, HTN:27.1, PU:28.6, OB:30.8, SMK:NR | Equation: MDRD Double: FALSE Method: FALSE Calibrated: TRUE Traceable: TRUE | 78% | Overall: 13.07% Male: NR Female: NR | 1.8 | 3.2 | 7.7 | 0.4 | 0.0 |
| Covic, A ([101](#_ENREF_101)) 01/2004 to 12/2005 | Romainia | n=19509, setting:MIXED, mean age:47.7, mean BMI:NR, 60.9% females. | DM:NR, HTN:NR, PU:NR, OB:NR, SMK:NR | Equation: MDRD Double: FALSE Method: TRUE Calibrated: TRUE Traceable: FALSE | 57% | Overall: 11.7% Male: 10% Female: 12.8% | NR | NR | 10.7 | 0.6 | 0.4 |
| Crews, D.C. ([120](#_ENREF_120)) 2004 to 2008 | USA | n=2375, setting:URBAN, mean age:48.3, mean BMI:NR, 49% females. | DM:16.7, HTN:45.4, PU:NR, OB:42.8, SMK:69.1 | Equation: MDRD Double: FALSE Method: TRUE Calibrated: TRUE Traceable: TRUE | 84% | Overall: 6.2% Male: NR Female: NR | NR | NR | NR | NR | NR |
| de Francisco, A.L.M. ([106](#_ENREF_106)) 2004 | Spain | n=7202, setting:MIXED, mean age:60.6, mean BMI:28.1, 52.7% females. | DM:31.5, HTN:66.7, PU:NR, OB:32.1, SMK:18.6 | Equation: MDRD Double: FALSE Method: FALSE Calibrated: FALSE Traceable: FALSE | 52% | Overall: 21.3% Male: 13.4% Female: 28.3% | NR | NR | 19.7 | 1.2 | 0.4 |
| Denburg, M.R ([117](#_ENREF_117)) 01/01/2002 to 2008 | UK | n=230426, setting:MIXED, mean age:NR, mean BMI:NR, NR females. | DM:NR, HTN:NR, PU:NR, OB:NR, SMK:NR | Equation: MDRD Double: FALSE Method: FALSE Calibrated: FALSE Traceable: FALSE | 47% | Overall: 4.32% Male: NR Female: NR | NR | NR | NR | NR | NR |
| Elorza-Ricard, J.M. ([107](#_ENREF_107)) 07/2008 to 06/2010 | Spain | n=447140, setting:URBAN, mean age:56.6, mean BMI:NR, 58.7% females. | DM:11, HTN:32.5, PU:NR, OB:NR, SMK:NR | Equation: MDRD Double: FALSE Method: TRUE Calibrated: FALSE Traceable: TRUE | 59% | Overall: 14.1% Male: NR Female: NR | NR | NR | 13.4 | 0.7 | 0.0 |
| Fleet Jamie, L ([76](#_ENREF_76)) 2007 to 2010 | Canada | n=123499, setting:MIXED, mean age:74, mean BMI:NR, 54% females. | DM:23, HTN:NR, PU:NR, OB:NR, SMK:NR | Equation: CKD-EPI Double: FALSE Method: FALSE Calibrated: FALSE Traceable: FALSE | 52% | Overall: 36.4% Male: NR Female: NR | NR | NR | 32.0 | 3.7 | 0.5 |
| Gallieni ([82](#_ENREF_82)) 10/2007 to 11/2008 | India | n=2536, setting:MIXED, mean age:51, mean BMI:20.2, 61% females. | DM:-99, HTN:39.4, PU:, OB:NR, SMK:11 | Equation: MDRD Double: FALSE Method: FALSE Calibrated: FALSE Traceable: FALSE | 57% | Overall: 4.2% Male: 4.7% Female: 3.7% | NR | NR | 3.5 | 0.7 | 0.0 |
| Gambaro, G ([54](#_ENREF_54)) 01/2006 | Italy | n=3629, setting:MIXED, mean age:59.8, mean BMI:NR, 52.2% females. | DM:NR, HTN:NR, PU:NR, OB:NR, SMK:NR | Equation: CKD-EPI Double: FALSE Method: TRUE Calibrated: TRUE Traceable: FALSE | 59% | Overall: 12.7% Male: 13.2% Female: 12.2% | 1.7 | 4.3 | 6.4 | 0.3 | 0.0 |
| Gao, S [Aboriginal] ([74](#_ENREF_74)) 07/2003 to 06/2004 | Australia | n=14989, setting:MIXED, mean age:NR, mean BMI:NR, NR females. | DM:NR, HTN:NR, PU:NR, OB:NR, SMK:NR | Equation: MDRD Double: FALSE Method: FALSE Calibrated: FALSE Traceable: FALSE | 40% | Overall: 6% Male: NR Female: NR | NR | NR | 5.1 | 0.6 | 0.3 |
| Gao, S [Non-Aboriginal] ([74](#_ENREF_74)) 07/2003 to 06/2004 | Australia | n=658664, setting:MIXED, mean age:NR, mean BMI:NR, NR females. | DM:NR, HTN:NR, PU:NR, OB:NR, SMK:NR | Equation: MDRD Double: FALSE Method: FALSE Calibrated: TRUE Traceable: TRUE | 57% | Overall: 6.8% Male: NR Female: NR | NR | NR | 6.3 | 0.4 | 0.1 |
| Giavarina, D. ([91](#_ENREF_91)) 05/2009 to 10/2009 | italy | n=39188, setting:MIXED, mean age:50.5, mean BMI:NR, 50% females. | DM:NR, HTN:NR, PU:NR, OB:NR, SMK:NR | Equation: MDRD Double: FALSE Method: TRUE Calibrated: TRUE Traceable: TRUE | 54% | Overall: 7.5% Male: NR Female: NR | NR | NR | 6.4 | 0.6 | 0.6 |
| Gifford, F.J. [1st Population] ([118](#_ENREF_118)) 2004 | UK | n=103322, setting:MIXED, mean age:61.5, mean BMI:NR, 57% females. | DM:NR, HTN:NR, PU:NR, OB:NR, SMK:NR | Equation: MDRD Double: FALSE Method: TRUE Calibrated: FALSE Traceable: TRUE | 57% | Overall: 5.4% Male: NR Female: NR | NR | NR | 5.1 | 0.3 | 0.0 |
| Gifford, F.J. [2nd Population] ([118](#_ENREF_118)) 04/2009 to 04/2010 | UK | n=123121, setting:MIXED, mean age:63.5, mean BMI:NR, 56% females. | DM:NR, HTN:NR, PU:NR, OB:NR, SMK:NR | Equation: MDRD Double: FALSE Method: TRUE Calibrated: FALSE Traceable: TRUE | 57% | Overall: 5.7% Male: NR Female: NR | NR | NR | 5.3 | 0.3 | 0.0 |
| Glynn, L.G ([89](#_ENREF_89)) 2008 | IRISH | n=2602, setting:MIXED, mean age:NR, mean BMI:NR, NR females. | DM:NR, HTN:NR, PU:NR, OB:NR, SMK:NR | Equation: MDRD Double: FALSE Method: FALSE Calibrated: FALSE Traceable: FALSE | 26% | Overall: 16.7% Male: NR Female: NR | NR | NR | 15.5 | 1.2 | 0.0 |
| Go, A.S ([2](#_ENREF_2)) 1996 to 2000 | USA | n=1120295, setting:MIXED, mean age:52.2, mean BMI:NR, 54.6% females. | DM:9.6, HTN:19.1, PU:6.3, OB:NR, SMK:NR | Equation: MDRD Double: FALSE Method: FALSE Calibrated: FALSE Traceable: TRUE | 65% | Overall: 17.5% Male: NR Female: NR | NR | NR | 16.8 | 0.6 | 0.1 |
| Gu, D-F. ([30](#_ENREF_30)) 2007 to 2009 | China | n=7801, setting:MIXED, mean age:43.9, mean BMI:24.92, 55.7% females. | DM:2.9, HTN:9.7, PU:NR, OB:NR, SMK:17.6 | Equation: MDRD Double: FALSE Method: FALSE Calibrated: FALSE Traceable: FALSE | 42% | Overall: 12.5% Male: NR Female: NR | NR | NR | NR | NR | NR |
| Hallan, S.I ([96](#_ENREF_96)) 1995 to 2004 | Norway | n=65604, setting:MIXED, mean age:49, mean BMI:NR, 53.2% females. | DM:3, HTN:11.1, PU:NR, OB:16.3, SMK:27.4 | Equation: MDRD Double: FALSE Method: TRUE Calibrated: TRUE Traceable: TRUE | 75% | Overall: 4.7% Male: NR Female: NR | NR | NR | NR | NR | NR |
| Hemmelgarn, BR ([77](#_ENREF_77)) 07/2001 to 07/2003 | Canada | n=10184, setting:MIXED, mean age:76, mean BMI:NR, 57.4% females. | DM:16.6, HTN:NR, PU:NR, OB:NR, SMK:NR | Equation: MDRD Double: FALSE Method: FALSE Calibrated: TRUE Traceable: FALSE | 63% | Overall: 35.4% Male: NR Female: NR | NR | NR | 31.3 | 4.1 | 0.0 |
| Hooi, L S ([73](#_ENREF_73)) 04/2011 to 07/2011 | West Malasia | n=876, setting:MIXED, mean age:42.9, mean BMI:NR, 52.1% females. | DM:19.6, HTN:38.4, PU:NR, OB:NR, SMK:19.8 | Equation: CKD-EPI Double: FALSE Method: TRUE Calibrated: TRUE Traceable: TRUE | 75% | Overall: 9.1% Male: NR Female: NR | 4.6 | 2.1 | 2.3 | 0.2 | 0.4 |
| Hosseinpanah, F. ([86](#_ENREF_86)) 1997 to 2000 | Iran | n=10063, setting:URBAN, mean age:42.7, mean BMI:NR, 58.0% females. | DM:13.7, HTN:25.8, PU:NR, OB:23.2, SMK:13.1 | Equation: MDRD Double: TRUE Method: TRUE Calibrated: TRUE Traceable: FALSE | 80% | Overall: 18.9% Male: NR Female: NR | NR | NR | 18.7 | 0.1 | 0.2 |
| Hsu, C. ([113](#_ENREF_113)) 2001 & 2002 | Taiwan | n=6001, setting:MIXED, mean age:NR, mean BMI:NR, NR females. | DM:NR, HTN:NR, PU:NR, OB:NR, SMK:NR | Equation: MDRD Double: FALSE Method: FALSE Calibrated: FALSE Traceable: FALSE | 47% | Overall: 6.9% Male: NR Female: NR | NR | NR | 6.5 | 0.3 | 0.1 |
| Huang, W.H. ([31](#_ENREF_31)) 2014 | China | n=3432, setting:URBAN, mean age:48.4, mean BMI:NR, 53.5% females. | DM:7.1, HTN:23.8, PU:6.09, OB:NR, SMK:NR | Equation: MDRD Double: FALSE Method: FALSE Calibrated: FALSE Traceable: FALSE | 21% | Overall: 9.5% Male: NR Female: NR | NR | NR | NR | NR | NR |
| Huda, M. N. ([47](#_ENREF_47)) 07/2003 to 06/2005 | Bangladesh | n=1000, setting:URBAN, mean age:NR, mean BMI:NR, 66.6% females. | DM:4.1, HTN:11.6, PU:7.7, OB:3, SMK:42.4 | Equation: MDRD Double: FALSE Method: TRUE Calibrated: FALSE Traceable: FALSE | 53% | Overall: 13.1% Male: NR Female: NR | 2.7 | 3.9 | 6.3 | 0.1 | 0.1 |
| Imai, E. ([56](#_ENREF_56)) 2000 to 2004 | Japan | n=574024, setting:MIXED, mean age:NR, mean BMI:NR, 58.1% females. | DM:NR, HTN:NR, PU:NR, OB:NR, SMK:NR | Equation: MDRD Double: FALSE Method: FALSE Calibrated: TRUE Traceable: TRUE | 57% | Overall: 12.9% Male: NR Female: NR | 0.6 | 1.7 | 10.4 | 0.1 | 0.1 |
| Iseki, K ([93](#_ENREF_93)) 04/2005 to 03/2006 | Japan | n=271013, setting:MIXED, mean age:NR, mean BMI:NR, 62.3% females. | DM:8.8, HTN:28.5, PU:NR, OB:NR, SMK:NR | Equation: MDRD Double: FALSE Method: TRUE Calibrated: TRUE Traceable: TRUE | 66% | Overall: 16.3% Male: 17.2% Female: 15.4% | NR | NR | NR | NR | NR |
| Jessani, S ([97](#_ENREF_97)) 2004 to 2005 | Pakistan | n=2873, setting:MIXED, mean age:51.5, mean BMI:25.8, 52.2% females. | DM:21.4, HTN:44.9, PU:NR, OB:NR, SMK:38.7 | Equation: CKD-EPI Double: FALSE Method: FALSE Calibrated: TRUE Traceable: FALSE | 65% | Overall: 12.5% Male: NR Female: NR | NR | NR | NR | NR | NR |
| Jiang, L. ([32](#_ENREF_32)) 2006 to 2007 | China | n=5105, setting:RURAL, mean age:52, mean BMI:NR, 55.0% females. | DM:6.6, HTN:40.5, PU:15.3, OB:NR, SMK:26 | Equation: MDRD Double: FALSE Method: FALSE Calibrated: FALSE Traceable: FALSE | 47% | Overall: 15.2% Male: 13.3% Female: 16.9% | 11.6 | 3.2 | 0.3 | 0.0 | 0.0 |
| Jose, M.D. ([115](#_ENREF_115)) 1995 to 2007 | Tasmania | n=223288, setting:MIXED, mean age:NR, mean BMI:NR, 54.0% females. | DM:NR, HTN:NR, PU:NR, OB:NR, SMK:NR | Equation: MDRD Double: TRUE Method: TRUE Calibrated: TRUE Traceable: TRUE | 88% | Overall: 11.6% Male: 10.1% Female: 13.1% | NR | NR | NR | NR | NR |
| Juutilainen, A [2002] ([79](#_ENREF_79)) 2002 | Finland | n=7049, setting:MIXED, mean age:47.6, mean BMI:26.9, NR females. | DM:4.6, HTN:39.4, PU:, OB:NR, SMK:27.6 | Equation: CKD-EPI Double: FALSE Method: FALSE Calibrated: TRUE Traceable: FALSE | 56% | Overall: 1.6% Male: 1.4% Female: 1.8% | NR | NR | 1.5 | 0.1 | 0.0 |
| Juutilainen, A [2007] ([79](#_ENREF_79)) 2007 | Finland | n=4228, setting:MIXED, mean age:49.7, mean BMI:27.1, NR females. | DM:8.4, HTN:42.6, PU:, OB:NR, SMK:20.6 | Equation: CKD-EPI Double: FALSE Method: FALSE Calibrated: TRUE Traceable: FALSE | 56% | Overall: 2.6% Male: 1.9% Female: 3.1% | NR | NR | 2.4 | 0.2 | 0.0 |
| Kang, H.T. [KHANES2] ([64](#_ENREF_64)) 2007 to 2009 | South Korea | n=5066, setting:MIXED, mean age:43.6, mean BMI:23.6, 56.8% females. | DM:6.4, HTN:22.2, PU:2, OB:31, SMK:23.2 | Equation: MDRD Double: FALSE Method: FALSE Calibrated: FALSE Traceable: FALSE | 47% | Overall: 9.2% Male: 7.9% Female: 11.3% | NR | NR | NR | NR | NR |
| Kang, H.T. [KHANES3] ([64](#_ENREF_64)) 2007 to 2009 | South Korea | n=5314, setting:MIXED, mean age:45.8, mean BMI:23.5, 57.6% females. | DM:7.3, HTN:26.3, PU:2.6, OB:30.4, SMK:28.5 | Equation: MDRD Double: FALSE Method: FALSE Calibrated: FALSE Traceable: FALSE | 47% | Overall: 9.8% Male: 5.4% Female: 12% | NR | NR | NR | NR | NR |
| Kang, H.T. [KHANES4] ([64](#_ENREF_64)) 2007 to 2009 | South Korea | n=15975, setting:MIXED, mean age:44.8, mean BMI:23.6, 57% females. | DM:7.5, HTN:24.1, PU:2.2, OB:31.3, SMK:23.4 | Equation: MDRD Double: FALSE Method: FALSE Calibrated: FALSE Traceable: FALSE | 47% | Overall: 5.5% Male: 4.5% Female: 6.3% | NR | NR | NR | NR | NR |
| Khajehdehi, P ([87](#_ENREF_87)) 2009 to 2012 | Iran | n=9404, setting:MIXED, mean age:39.8, mean BMI:NR, 64.1% females. | DM:NR, HTN:NR, PU:NR, OB:NR, SMK:NR | Equation: MDRD Double: FALSE Method: FALSE Calibrated: FALSE Traceable: FALSE | 38% | Overall: 11.4% Male: 5.2% Female: 14.8% | NR | NR | 11.2 | 0.1 | 0.1 |
| Kim, S. ([65](#_ENREF_65)) 2006 | South Korea | n=2356, setting:URBAN, mean age:50.5, mean BMI:NR, 51% females. | DM:10.1, HTN:35.5, PU:10.2, OB:NR, SMK:23 | Equation: MDRD Double: FALSE Method: TRUE Calibrated: FALSE Traceable: FALSE | 60% | Overall: 13.7% Male: NR Female: NR | 2.0 | 6.7 | 0.5 | 0.2 | 0.0 |
| Kramer, H [White] ([44](#_ENREF_44)) 08/2000 to 07/2002 | USA | n=2594, setting:MIXED, mean age:63.1, mean BMI:27.8, 51.9% females. | DM:8, HTN:38.2, PU:NR, OB:NR, SMK:11.6 | Equation: MDRD Double: FALSE Method: TRUE Calibrated: TRUE Traceable: FALSE | 69% | Overall: 13.6% Male: 9.9% Female: 17.3% | NR | NR | NR | NR | NR |
| Kramer, H [Black] ([44](#_ENREF_44)) 08/2000 to 07/2002 | USA | n=1872, setting:MIXED, mean age:62.8, mean BMI:30, 55.2% females. | DM:20.4, HTN:59.9, PU:NR, OB:NR, SMK:18 | Equation: MDRD Double: FALSE Method: TRUE Calibrated: TRUE Traceable: FALSE | 69% | Overall: 7.55% Male: 7.9% Female: 7.2% | NR | NR | NR | NR | NR |
| Kramer, H [Chinese] ([44](#_ENREF_44)) 08/2000 to 07/2002 | USA | n=778, setting:MIXED, mean age:63, mean BMI:24, 53.1% females. | DM:15.3, HTN:38.7, PU:NR, OB:NR, SMK:5.35 | Equation: MDRD Double: FALSE Method: TRUE Calibrated: TRUE Traceable: FALSE | 69% | Overall: 10.25% Male: 9.8% Female: 10.7% | NR | NR | NR | NR | NR |
| Kramer, H [Hispanic] ([44](#_ENREF_44)) 08/2000 to 07/2002 | USA | n=1486, setting:MIXED, mean age:61.8, mean BMI:29.4, 51.7% females. | DM:12.6, HTN:41.4, PU:NR, OB:NR, SMK:13.7 | Equation: MDRD Double: FALSE Method: TRUE Calibrated: TRUE Traceable: FALSE | 69% | Overall: 8.25% Male: 6.9% Female: 9.6% | NR | NR | NR | NR | NR |
| Li, Z-Y. ([33](#_ENREF_33)) NR | China | n=2310, setting:URBAN, mean age:60.7, mean BMI:23.6, 50.5% females. | DM:28.5, HTN:47.1, PU:4.7, OB:4.55, SMK:NR | Equation: MDRD Double: FALSE Method: TRUE Calibrated: FALSE Traceable: FALSE | 44% | Overall: 12.9% Male: NR Female: NR | NR | NR | NR | NR | NR |
| Liang, Y ([34](#_ENREF_34)) 06/2010 to 09/2012 | China | n=10926, setting:MIXED, mean age:43.3, mean BMI:24.5, 37.7% females. | DM:NR, HTN:NR, PU:, OB:5.1, SMK:NR | Equation: CKD-EPI Double: FALSE Method: FALSE Calibrated: FALSE Traceable: FALSE | 33% | Overall: 5.9% Male: NR Female: NR | NR | NR | NR | NR | NR |
| Lin, B ([35](#_ENREF_35)) 2009 to 2012 | China | n=10384, setting:MIXED, mean age:52.9, mean BMI:23.1, 57% females. | DM:NR, HTN:NR, PU:8.65, OB:NR, SMK:NR | Equation: MDRD Double: FALSE Method: TRUE Calibrated: FALSE Traceable: FALSE | 53% | Overall: 9.88% Male: NR Female: NR | 5.0 | 3.1 | 1.7 | 0.1 | 0.0 |
| Liu, Q. ([36](#_ENREF_36)) 2006 to 2007 | China | n=1186, setting:RURAL, mean age:49.4, mean BMI:23, NR females. | DM:4.5, HTN:22, PU:7.1, OB:NR, SMK:29.3 | Equation: MDRD Double: FALSE Method: TRUE Calibrated: FALSE Traceable: FALSE | 58% | Overall: 13.6% Male: NR Female: NR | NR | NR | NR | NR | NR |
| Lou Arnal, L.M. ([108](#_ENREF_108)) 2006 | Spain | n=18922, setting:MIXED, mean age:60, mean BMI:NR, 57.1% females. | DM:NR, HTN:NR, PU:NR, OB:NR, SMK:NR | Equation: MDRD Double: FALSE Method: TRUE Calibrated: FALSE Traceable: FALSE | 46% | Overall: 16.4% Male: 12.9% Female: 19.1% | NR | NR | 15.7 | 0.6 | 0.1 |
| Lu, C ([37](#_ENREF_37)) 06/2007 to 01/2009 | China | n=2576, setting:URBAN, mean age:51, mean BMI:24.7, 47.4% females. | DM:4.8, HTN:32.3, PU:NR, OB:NR, SMK:14.5 | Equation: MDRD Double: FALSE Method: FALSE Calibrated: TRUE Traceable: FALSE | 56% | Overall: 9.8% Male: 9.5% Female: 10.1% | 4.9 | 2.7 | 2.2 | 0.0 | 0.0 |
| Maple-Brown, L.J ([45](#_ENREF_45)) 09/2003 to 03/2005 | Australia | n=860, setting:URBAN, mean age:35.3, mean BMI:28.3, 68.1% females. | DM:NR, HTN:NR, PU:14.8, OB:NR, SMK:NR | Equation: MDRD Double: FALSE Method: TRUE Calibrated: TRUE Traceable: TRUE | 77% | Overall: 16.5% Male: NR Female: NR | NR | NR | NR | NR | NR |
| Matsha, T.E. ([103](#_ENREF_103)) 2008 to 2011 | South Africa | n=1202, setting:URBAN, mean age:52.9, mean BMI:29.9, 75.3% females. | DM:26.4, HTN:NR, PU:NR, OB:NR, SMK:40.5 | Equation: MDRD Double: FALSE Method: FALSE Calibrated: FALSE Traceable: FALSE | 50% | Overall: 7.6% Male: 5% Female: 8.5% | NR | NR | NR | NR | NR |
| Minutolo, R ([92](#_ENREF_92)) 2003 | Italy | n=77630, setting:MIXED, mean age:NR, mean BMI:NR, 52.8% females. | DM:NR, HTN:NR, PU:NR, OB:NR, SMK:NR | Equation: MDRD Double: FALSE Method: FALSE Calibrated: FALSE Traceable: FALSE | 54% | Overall: 9.2% Male: 6.5% Female: 11.9% | NR | NR | NR | NR | NR |
| Nagata, M. ([94](#_ENREF_94)) 2002 | Japan | n=3297, setting:URBAN, mean age:61.6, mean BMI:NR, 57% females. | DM:15.4, HTN:36.1, PU:7.5, OB:26.2, SMK:24.9 | Equation: MDRD Double: FALSE Method: TRUE Calibrated: TRUE Traceable: TRUE | 73% | Overall: 18.2% Male: 22.1% Female: 15.3% | NR | NR | NR | NR | NR |
| Najafi, I. ([52](#_ENREF_52)) 2007 to 2009 | Iran | n=1557, setting:URBAN, mean age:56.8, mean BMI:NR, 53% females. | DM:26.6, HTN:50.8, PU:5.7, OB:27, SMK:15.63 | Equation: MDRD Double: FALSE Method: FALSE Calibrated: FALSE Traceable: FALSE | 40% | Overall: 23.4% Male: 23.5% Female: 22.9% | 3.8 | 10.7 | 8.4 | 0.3 | 0.3 |
| Nitsch, D. ([112](#_ENREF_112)) 2002 to 2003 | Swiss | n=6317, setting:MIXED, mean age:52.2, mean BMI:25.9, 50.9% females. | DM:3.5, HTN:39.3, PU:NR, OB:15.8, SMK:24.9 | Equation: MDRD Double: FALSE Method: TRUE Calibrated: TRUE Traceable: FALSE | 57% | Overall: 10.8% Male: NR Female: NR | NR | NR | NR | NR | NR |
| Nomura, I ([57](#_ENREF_57)) 09/2006 to 10/2006 | Japan | n=1978, setting:MIXED, mean age:60.8, mean BMI:21.6, 64.8% females. | DM:NR, HTN:NR, PU:NR, OB:NR, SMK:NR | Equation: MDRD Double: FALSE Method: FALSE Calibrated: FALSE Traceable: FALSE | 42% | Overall: 18.2% Male: 22.8% Female: 15.7% | NR | NR | NR | NR | NR |
| Nongpiur, M.E ([62](#_ENREF_62)) 08/2004 to July 2006 | Singapore | n=3280, setting:MIXED, mean age:58.7, mean BMI:26.4, 51.7% females. | DM:23.2, HTN:68.5, PU:NR, OB:NR, SMK:20.4 | Equation: MDRD Double: FALSE Method: FALSE Calibrated: FALSE Traceable: FALSE | 57% | Overall: 27.9% Male: NR Female: NR | NR | NR | NR | NR | 0.0 |
| O'Callaghan ([10](#_ENREF_10)) 10/2009 to 01/2011 | UK | n=660000, setting:MIXED, mean age:NR, mean BMI:NR, NR females. | DM:NR, HTN:NR, PU:NR, OB:NR, SMK:NR | Equation: MDRD Double: FALSE Method: TRUE Calibrated: TRUE Traceable: TRUE | 75% | Overall: 15.7% Male: NR Female: NR | NR | NR | 14.5 | 1.1 | 0.2 |
| Ohno, Y. ([58](#_ENREF_58)) 04/2001 to 03/2002 | Japan | n=39211, setting:MIXED, mean age:58.6, mean BMI:NR, 70.3% females. | DM:1.8, HTN:22.9, PU:NR, OB:21.2, SMK:NR | Equation: MDRD Double: FALSE Method: FALSE Calibrated: FALSE Traceable: FALSE | 50% | Overall: 20.5% Male: 21.7% Female: 20% | NR | NR | NR | NR | NR |
| Ong-Ajyooth, L. ([69](#_ENREF_69)) 2004 | Thailand | n=3117, setting:MIXED, mean age:33.6, mean BMI:23.2, 50.0% females. | DM:7, HTN:22.5, PU:NR, OB:NR, SMK:24.3 | Equation: MDRD Double: FALSE Method: TRUE Calibrated: FALSE Traceable: FALSE | 51% | Overall: 8.9% Male: 5.5% Female: 12.3% | NR | NR | 8.1 | 0.2 | 0.2 |
| Otero, A. ([109](#_ENREF_109)) NR | Spain | n=1059, setting:MIXED, mean age:50.5, mean BMI:NR, 51.4% females. | DM:7.5, HTN:31.5, PU:NR, OB:21.9, SMK:24.3 | Equation: MDRD Double: FALSE Method: FALSE Calibrated: FALSE Traceable: FALSE | 42% | Overall: 13.1% Male: NR Female: NR | NR | NR | 12.4 | 0.7 | 0.0 |
| Pani, A ([55](#_ENREF_55)) 2010 | Italy | n=4842, setting:MIXED, mean age:43.7, mean BMI:25.9, 58% females. | DM:9.1, HTN:32, PU:12.9, OB:18.1, SMK:23.7 | Equation: MDRD Double: FALSE Method: TRUE Calibrated: TRUE Traceable: FALSE | 59% | Overall: 15.2% Male: 12.9% Female: 15.4% | 5.5 | 5.5 | 4.0 | 0.0 | 0.0 |
| Pena Porta, JJ.M. ([110](#_ENREF_110)) 2007 | Spain | n=3286, setting:MIXED, mean age:NR, mean BMI:NR, 56.6% females. | DM:NR, HTN:NR, PU:NR, OB:NR, SMK:NR | Equation: MDRD Double: FALSE Method: TRUE Calibrated: FALSE Traceable: FALSE | 51% | Overall: 21.2% Male: 20% Female: 22% | NR | NR | 19.3 | 1.7 | 0.2 |
| Peralta, C. A [Black] ([121](#_ENREF_121)) 2005 to 2006 | USA | n=1898, setting:MIXED, mean age:46, mean BMI:28, 53% females. | DM:9, HTN:12, PU:NR, OB:NR, SMK:41 | Equation: MDRD Double: FALSE Method: FALSE Calibrated: TRUE Traceable: FALSE | 63% | Overall: 3.7% Male: NR Female: NR | NR | NR | 3.6 | 0.1 | 0.0 |
| Peralta, C. A. [White] ([121](#_ENREF_121)) 2005 to 2006 | USA | n=1651, setting:MIXED, mean age:45, mean BMI:31, 61% females. | DM:14, HTN:30, PU:NR, OB:NR, SMK:40 | Equation: MDRD Double: FALSE Method: FALSE Calibrated: TRUE Traceable: FALSE | 63% | Overall: 1.9% Male: NR Female: NR | NR | NR | 1.3 | 0.6 | 0.0 |
| Perkovic, V ([114](#_ENREF_114)) 2000 | Taiwan | n=5146, setting:MIXED, mean age:50.5, mean BMI:24.2, 51% females. | DM:10.2, HTN:18.9, PU:NR, OB:NR, SMK:23.8 | Equation: MDRD Double: FALSE Method: FALSE Calibrated: TRUE Traceable: FALSE | 44% | Overall: 16.3% Male: NR Female: NR | NR | NR | NR | NR | NR |
| Ponte, B ([66](#_ENREF_66)) 2003 to 2006 | Switzerland | n=5921, setting:MIXED, mean age:53, mean BMI:25.8, 52.5% females. | DM:6.6, HTN:35.8, PU:7.3, OB:15.6, SMK:26.9 | Equation: MDRD Double: FALSE Method: TRUE Calibrated: FALSE Traceable: TRUE | 71% | Overall: 13.8% Male: NR Female: NR | 1.5 | 3.9 | 8.3 | 0.6 | 0.1 |
| Prodjosundjadi, W ([84](#_ENREF_84)) NR | Indonesia | n=9412, setting:URBAN, mean age:43.3, mean BMI:NR, 64.1% females. | DM:3.5, HTN:15, PU:3, OB:32.5, SMK:19.8 | Equation: MDRD Double: FALSE Method: TRUE Calibrated: FALSE Traceable: FALSE | 51% | Overall: 7.5% Male: NR Female: NR | NR | NR | 6.0 | 0.7 | 0.9 |
| Robles N.R. ([111](#_ENREF_111)) 2009 to 2010 | Spain | n=2831, setting:MIXED, mean age:51.2, mean BMI:28.6, 53.5% females. | DM:14.1, HTN:39.6, PU:NR, OB:34.9, SMK:53.9 | Equation: MDRD Double: FALSE Method: TRUE Calibrated: FALSE Traceable: TRUE | 67% | Overall: 4% Male: NR Female: NR | NR | NR | 3.7 | 0.3 | 0.0 |
| Rothenbacher, D ([50](#_ENREF_50)) 03/23009 to 04/2010 | German | n=1471, setting:MIXED, mean age:75.6, mean BMI:27.6, 43.2% females. | DM:13.4, HTN:53.3, PU:NR, OB:24.2, SMK:9.3 | Equation: MDRD Double: FALSE Method: TRUE Calibrated: FALSE Traceable: TRUE | 69% | Overall: 34.3% Male: 36.4% Female: 42.1% | 0.3 | 4.3 | 32.9 | 1.4 | 0.0 |
| Ruthowski, B. ([99](#_ENREF_99)) 2004 to 2005 | Poland | n=2476, setting:MIXED, mean age:50, mean BMI:26.6, 61.8% females. | DM:6.7, HTN:29.3, PU:15.5, OB:NR, SMK:NR | Equation: MDRD Double: FALSE Method: TRUE Calibrated: FALSE Traceable: FALSE | 51% | Overall: 18.4% Male: NR Female: NR | NR | NR | 8.1 | 0.6 | 0.0 |
| Ryan, T..P ([122](#_ENREF_122)) 01/2003 to 05/2005 | USA | n=24492, setting:MIXED, mean age:56.2, mean BMI:NR, 56% females. | DM:NR, HTN:NR, PU:NR, OB:NR, SMK:NR | Equation: MDRD Double: TRUE Method: TRUE Calibrated: TRUE Traceable: TRUE | 84% | Overall: 28.3% Male: NR Female: NR | NR | NR | 24.5 | 2.9 | 0.9 |
| Sabanayagam, C ([63](#_ENREF_63)) NR | Singapore | n=4499, setting:MIXED, mean age:49.7, mean BMI:24.0, 52.6% females. | DM:11.0, HTN:40.4, PU:NR, OB:NR, SMK:11.6 | Equation: MDRD Double: FALSE Method: TRUE Calibrated: TRUE Traceable: TRUE | 73% | Overall: 15.6% Male: NR Female: NR | 4.3 | 5.7 | 5.3 | 0.2 | 0.0 |
| Safinejad, M.R. ([53](#_ENREF_53)) 2002 to 2005 | Iran | n=16354, setting:MIXED, mean age:51, mean BMI:NR, 51% females. | DM:NR, HTN:NR, PU:NR, OB:NR, SMK:NR | Equation: MDRD Double: FALSE Method: TRUE Calibrated: FALSE Traceable: FALSE | 56% | Overall: 12.6% Male: 14.1% Female: 11.3% | 2.2 | 2.1 | 7.8 | 0.3 | 0.2 |
| Sahin, I. ([116](#_ENREF_116)) 2005 | Turkey | n=1079, setting:MIXED, mean age:41.4, mean BMI:26.8, 50.6% females. | DM:5.28, HTN:37.8, PU:NR, OB:22.9, SMK:NR | Equation: MDRD Double: FALSE Method: FALSE Calibrated: FALSE Traceable: FALSE | 28% | Overall: 5.8% Male: NR Female: NR | NR | NR | 5.1 | 0.7 | 0.0 |
| Seck, S.M. ([61](#_ENREF_61)) 2012 | Senegal | n=1037, setting:MIXED, mean age:48, mean BMI:26.3, 60% females. | DM:12.7, HTN:39.1, PU:5.3, OB:23.4, SMK:4.2 | Equation: MDRD Double: FALSE Method: TRUE Calibrated: FALSE Traceable: FALSE | 44% | Overall: 4.9% Male: 6.9% Female: 5.7% | NR | NR | NR | NR | NR |
| Shan, Y. ([38](#_ENREF_38)) 05/2007 to 03/2008 | China | n=3981, setting:URBAN, mean age:51.7, mean BMI:23.3, 45.3% females. | DM:5.8, HTN:15.04, PU:5, OB:NR, SMK:14.4 | Equation: MDRD Double: FALSE Method: TRUE Calibrated: FALSE Traceable: FALSE | 60% | Overall: 10.5% Male: 8.17% Female: 13.5% | 6.7 | 2.9 | 0.9 | 0.1 | 0.0 |
| Sharma, S.K. [2013] ([59](#_ENREF_59)) 2003 to 2005 | Nepal | n=3218, setting:URBAN, mean age:42.9, mean BMI:NR, 52.1% females. | DM:7.5, HTN:38.6, PU:5.1, OB:5, SMK:23.7 | Equation: MDRD Double: FALSE Method: FALSE Calibrated: FALSE Traceable: FALSE | 33% | Overall: 10.6% Male: NR Female: NR | NR | NR | NR | NR | NR |
| Sharma, S.K. [China] ([39](#_ENREF_39)) 2007 | China | n=1999, setting:MIXED, mean age:53, mean BMI:23.1, 69.7% females. | DM:5.5, HTN:30, PU:2.8, OB:NR, SMK:11 | Equation: MDRD Double: FALSE Method: FALSE Calibrated: FALSE Traceable: FALSE | 42% | Overall: 14.6% Male: 14.6% Female: 13.7% | NR | NR | 14.0 | 0.3 | 0.3 |
| Sharma, S.K. [Mongolia] ([39](#_ENREF_39)) 2007 | Mongolia | n=997, setting:MIXED, mean age:40, mean BMI:25, 74% females. | DM:2.2, HTN:24, PU:5.4, OB:NR, SMK:17 | Equation: MDRD Double: FALSE Method: FALSE Calibrated: FALSE Traceable: FALSE | 42% | Overall: 8.07% Male: 5.1% Female: 9.6% | NR | NR | 7.0 | 1.0 | 0.1 |
| Sharma, S.K. [Nepal] ([39](#_ENREF_39)) 2007 | Nepal | n=8398, setting:MIXED, mean age:38, mean BMI:22.8, 62% females. | DM:7.7, HTN:34, PU:10.3, OB:NR, SMK:11 | Equation: MDRD Double: FALSE Method: FALSE Calibrated: FALSE Traceable: FALSE | 42% | Overall: 14.4% Male: 11.4% Female: 16.4% | NR | NR | 14.0 | 0.3 | 0.1 |
| Shin, S-Y ([104](#_ENREF_104)) 2010 | South Korea | n=5286, setting:MIXED, mean age:NR, mean BMI:23.7, 54.6% females. | DM:8.4, HTN:22.9, PU:7.9, OB:32.1, SMK:21.8 | Equation: MDRD Double: FALSE Method: TRUE Calibrated: FALSE Traceable: TRUE | 74% | Overall: 3% Male: NR Female: NR | NR | NR | NR | NR | NR |
| Singh, N.P. ([83](#_ENREF_83)) 2005 to 2007 | India | n=5252, setting:MIXED, mean age:38.9, mean BMI:23.2, 39.9% females. | DM:6.51, HTN:27.74, PU:2.25, OB:23.6, SMK:19.9 | Equation: MDRD Double: FALSE Method: TRUE Calibrated: FALSE Traceable: FALSE | 58% | Overall: 4.2% Male: 2.7% Female: 6.3% | NR | NR | 3.8 | 0.2 | 0.2 |
| Stengel, B ([80](#_ENREF_80)) 1999 to 2001 | France | n=8705, setting:MIXED, mean age:74.3, mean BMI:NR, 60.5% females. | DM:9.7, HTN:77.3, PU:NR, OB:13.2, SMK:5.6 | Equation: MDRD Double: FALSE Method: TRUE Calibrated: FALSE Traceable: TRUE | 74% | Overall: 13.7% Male: NR Female: NR | NR | NR | 13.4 | 0.3 | 0.0 |
| Stevens P.E. ([119](#_ENREF_119)) 1998 to 2003 | UK | n=38262, setting:MIXED, mean age:58.1, mean BMI:27.1, 56.5% females. | DM:10.6, HTN:55.8, PU:12.5, OB:NR, SMK:NR | Equation: MDRD Double: FALSE Method: TRUE Calibrated: TRUE Traceable: FALSE | 62% | Overall: 8.5% Male: 5.8% Female: 10.6% | NR | NR | NR | NR | NR |
| Suleymanalar, G. ([71](#_ENREF_71)) 2006 to 2009 | Turkey | n=10748, setting:MIXED, mean age:40.5, mean BMI:NR, 56% females. | DM:12.7, HTN:32.7, PU:12.2, OB:20.1, SMK:NR | Equation: MDRD Double: FALSE Method: FALSE Calibrated: FALSE Traceable: FALSE | 52% | Overall: 15.7% Male: 12.8% Female: 18.4% | 5.4 | 5.2 | 4.7 | 0.3 | 0.2 |
| Sumaili, E.K. ([49](#_ENREF_49)) 2007 to 2009 | Congo | n=4460, setting:MIXED, mean age:NR, mean BMI:NR, NR females. | DM:7.7, HTN:39.5, PU:11.5, OB:12.5, SMK:NR | Equation: MDRD Double: FALSE Method: FALSE Calibrated: FALSE Traceable: FALSE | 35% | Overall: 12.4% Male: NR Female: NR | NR | NR | NR | NR | NR |
| Tanaka, H ([95](#_ENREF_95)) 05/2003 to 03/2004 | Japan | n=6980, setting:MIXED, mean age:49.6, mean BMI:24.2, 45.1% females. | DM:14.3, HTN:45.2, PU:NR, OB:12, SMK:NR | Equation: MDRD Double: FALSE Method: FALSE Calibrated: FALSE Traceable: FALSE | 50% | Overall: 13.7% Male: NR Female: NR | NR | NR | NR | NR | NR |
| Tanner, R.M. [Black] ([123](#_ENREF_123)) 06/2003 tp 10/2007 | USA | n=11109, setting:MIXED, mean age:59, mean BMI:NR, 55.6% females. | DM:24.3, HTN:64.2, PU:, OB:47.4, SMK:NR | Equation: CKD-EPI Double: FALSE Method: TRUE Calibrated: FALSE Traceable: TRUE | 74% | Overall: 9.4% Male: NR Female: NR | NR | NR | NR | NR | NR |
| Tanner, R.M. [White] ([123](#_ENREF_123)) 06/2003 tp 10/2007 | USA | n=16410, setting:MIXED, mean age:61.4, mean BMI:NR, 52.3% females. | DM:11.8, HTN:41.4, PU:, OB:31.2, SMK:NR | Equation: CKD-EPI Double: FALSE Method: TRUE Calibrated: FALSE Traceable: TRUE | 74% | Overall: 9.2% Male: NR Female: NR | NR | NR | NR | NR | NR |
| Tomonaga, Y. ([67](#_ENREF_67)) 2010 | Switzerland | n=1000, setting:MIXED, mean age:57, mean BMI:27.5, 57% females. | DM:NR, HTN:NR, PU:17.1, OB:NR, SMK:17.5 | Equation: CKD-EPI Double: FALSE Method: TRUE Calibrated: FALSE Traceable: FALSE | 53% | Overall: 23% Male: NR Female: NR | 6.0 | 6.6 | 10.4 | 0.0 | 0.0 |
| Van Bilijderveen, J.C. ([60](#_ENREF_60)) 01/1996 to 03/2011 | Netherlands | n=784563, setting:MIXED, mean age:45.5, mean BMI:NR, 51.9% females. | DM:4.3, HTN:NR, PU:NR, OB:NR, SMK:NR | Equation: CKD-EPI Double: FALSE Method: FALSE Calibrated: FALSE Traceable: FALSE | 38% | Overall: 5.1% Male: NR Female: NR | 0.3 | 0.0 | 4.2 | 0.4 | 0.2 |
| Van Pottelbergh ([75](#_ENREF_75)) 1994 to 2008 | Belgium | n=34642, setting:MIXED, mean age:69, mean BMI:NR, 53.8% females. | DM:NR, HTN:NR, PU:NR, OB:NR, SMK:NR | Equation: MDRD Double: TRUE Method: FALSE Calibrated: FALSE Traceable: FALSE | 44% | Overall: 23.9% Male: NR Female: NR | NR | NR | 20.6 | 2.3 | 0.0 |
| Vinhas, J. ([100](#_ENREF_100)) 2008 to 2009 | Portugal | n=5167, setting:MIXED, mean age:56.2, mean BMI:NR, 59.8% females. | DM:11.7, HTN:44.8, PU:NR, OB:33.7, SMK:NR | Equation: MDRD Double: FALSE Method: TRUE Calibrated: FALSE Traceable: FALSE | 46% | Overall: 6.1% Male: 3.7% Female: 7.8% | NR | NR | 5.6 | 0.3 | 0.2 |
| Wen, C.P. ([68](#_ENREF_68)) 1994 to 2006 | Taiwan | n=462293, setting:MIXED, mean age:41.8, mean BMI:23.2, 50.2% females. | DM:5.4, HTN:20.7, PU:7.9, OB:3.9, SMK:24.2 | Equation: MDRD Double: FALSE Method: TRUE Calibrated: FALSE Traceable: FALSE | 70% | Overall: 11.93% Male: 12.9% Female: 11% | 1.0 | 3.8 | 6.8 | 0.2 | 0.1 |
| White, S ([46](#_ENREF_46)) 1999 to 2000 | Australia | n=11247, setting:MIXED, mean age:51.5, mean BMI:NR, 54.8% females. | DM:8.5, HTN:NR, PU:NR, OB:NR, SMK:NR | Equation: MDRD Double: FALSE Method: TRUE Calibrated: TRUE Traceable: FALSE | 71% | Overall: 13.4% Male: 11.8% Female: 14.9% | 1.4 | 4.3 | 7.5 | 0.3 | 0.0 |
| Xu, G ([40](#_ENREF_40)) 01/2008 to 06/2011 | China | n=5708, setting:URBAN, mean age:46.8, mean BMI:25, 23.5% females. | DM:10.1, HTN:17.3, PU:25, OB:NR, SMK:35.5 | Equation: MDRD Double: FALSE Method: FALSE Calibrated: FALSE Traceable: FALSE | 47% | Overall: 1.1% Male: NR Female: NR | NR | NR | 1.1 | 0.0 | 0.0 |
| Zhang, L [2007] ([41](#_ENREF_41)) NR | China | n=2353, setting:URBAN, mean age:60.7, mean BMI:NR, 50.4% females. | DM:NR, HTN:NR, PU:6.2, OB:NR, SMK:11.9 | Equation: MDRD Double: FALSE Method: TRUE Calibrated: FALSE Traceable: FALSE | 58% | Overall: 11.3% Male: NR Female: NR | 1.9 | 4.3 | 5.2 | 0.0 | 0.0 |
| Zhang, L [2012] ([42](#_ENREF_42)) 2009 to 2010 | China | n=47204, setting:Mixed, mean age:49.6, mean BMI:23.9, 57.3% females. | DM:7.4, HTN:35.4, PU:6.6, OB:NR, SMK:23.5 | Equation: MDRD Double: FALSE Method: TRUE Calibrated: TRUE Traceable: FALSE | 71% | Overall: 10.8% Male: NR Female: NR | 5.7 | 3.4 | 1.6 | 0.1 | 0.0 |
| Zhang, L. [2008] ([43](#_ENREF_43)) 2006 | China | n=13925, setting:MIXED, mean age:40, mean BMI:NR, 43.9% females. | DM:6.3, HTN:30.2, PU:9.2, OB:38.5, SMK:32.9 | Equation: MDRD Double: FALSE Method: FALSE Calibrated: TRUE Traceable: FALSE | 63% | Overall: 14% Male: NR Female: NR | 7.4 | 4.7 | 1.8 | 0.0 | 0.0 |
| Zhang, Q-L. ([51](#_ENREF_51)) July 2000 to December 2002 | Germany | n=9806, setting:MIXED, mean age:62.1, mean BMI:NR, 55% females. | DM:11.6, HTN:44.2, PU:NR, OB:26.9, SMK:16.9 | Equation: MDRD Double: FALSE Method: TRUE Calibrated: FALSE Traceable: FALSE | 63% | Overall: 26.7% Male: NR Female: NR | 4.6 | 4.7 | 17.0 | 0.4 | 0.0 |
